# Measuring the gap: correlating synthetic-to-real drift with PHI de-identification performance

**DOI:** 10.1186/s44342-026-00072-9

**Published:** 2026-04-30

**Authors:** Joseph Cornelius, Fabio Rinaldi

**Affiliations:** 1 Dalle Molle Institute for Artificial Intelligence Research (IDSIA USI-SUPSI), Via la Santa 1, Lugano-Viganello, CH-6962 Ticino Switzerland; 2 Department of Quantitative Biomedicine, University of Zurich, Schmelzbergstrasse 26, Zurich, 8006 Switzerland

**Keywords:** Clinical text de-identification, Synthetic clinical data, Large language models, Distributional drift

## Abstract

Clinical text de-identification enables the use of electronic health records while protecting patient privacy, but public training data remain scarce and often have mismatched documentation styles. Recent works have proposed using large language models (LLMs) to generate synthetic clinical notes, but it remains unclear if they reflect distributions of real clinical notes. We examine how lexical and semantic drift across training and evaluation corpora affects protected health information (PHI) tagger performance. We generated synthetic notes from scratch for four categories using five generator LLMs and one judge LLM. Next, we fine-tuned small de-identification models on real, synthetic, and mixed corpora, and evaluated them on three external benchmarks under a harmonized label schema. Models trained on broad, clinically oriented sources transfer better than those on legal or narrowly synthetic data. These results suggest that although synthetic data lacks some real-world distributional properties, it remains useful in low-resource settings. We found that compact distributional and embedding-based drift measures moderately correlate with out-of-distribution F1 score, a practically important result because drift estimation can improve synthetic-data quality control and alignment.

## Introduction

Effective de-identification of protected health information (PHI) in clinical text is essential for privacy-preserving research. Yet access to public high-quality training data is often limited and remains severely constrained by regulatory and ethical barriers [1–3]. Although large language models (LLMs) are increasingly used for clinical text generation, many healthcare institutions with limited compute still rely on smaller pretrained language models (PLMs) for tasks like PHI de-identification to enable full on-premise training and deployment, which regulations set by the Health Insurance Portability and Accountability Act (HIPAA) in the US and the General Data Protection Regulation (GDPR) in the EU require [2, 4–6].

Synthetic clinical notes generated by LLMs to train smaller models offer a scalable, privacy-compliant alternative to real patient records and can be used off-premise [4, 7, 8]. They can be generated in multiple languages, annotated consistently, and tailored to specific task requirements without exposing sensitive patient information.

However, while some studies have suggested that synthetic clinical text can approximate the distributional properties of real notes to a useful degree [7, 8], others have argued that important institution- and task-specific nuances, stylistic and distributional characteristics are not adequately captured [9–12]. Several studies have further reported noticeable drops in performance when models trained on synthetic data are evaluated on real clinical data [9, 13, 14].

**Fig. 1:**
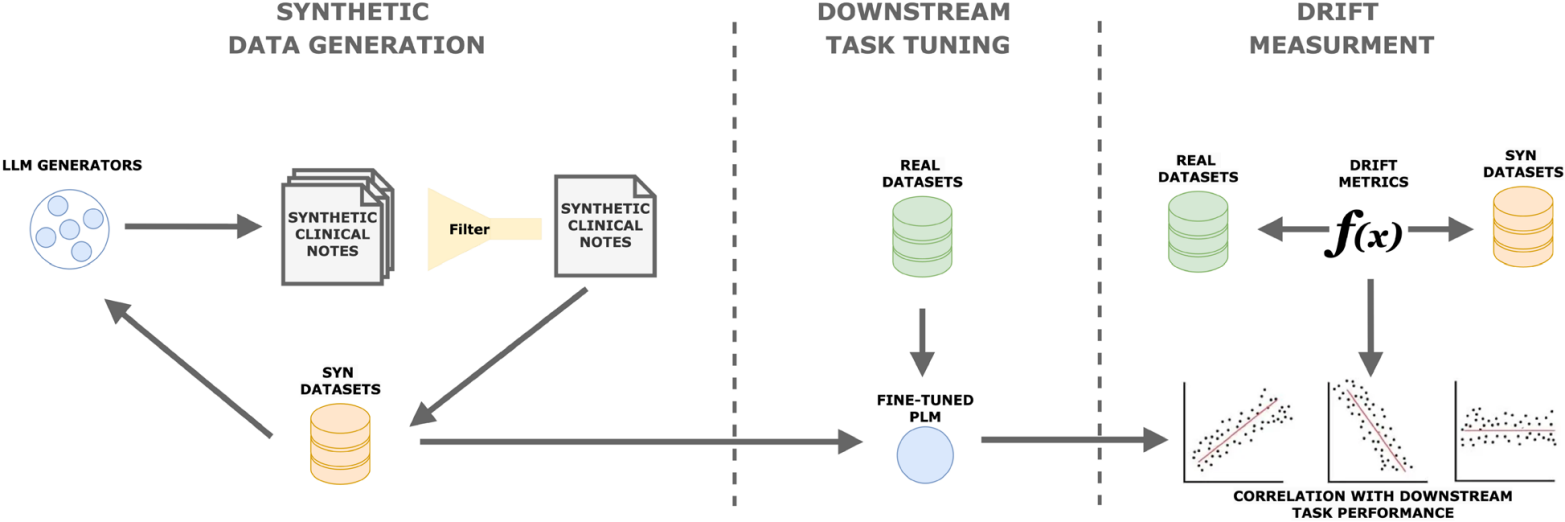
Pipeline overview. (1) LLM-based generation and quality filtering of synthetic notes; (2) fine-tuning of pretrained language models on downstream tasks with real and synthetic data; (3) computing dataset-drift metrics and correlating them with task performance

To gain better insight into the differences between synthetic and real clinical datasets, we conduct a preliminary analysis of selected lexical, structural, and semantic similarity measures in the context of the PHI de-identification task (Fig. 1).

We generated a synthetic clinical text corpus covering four PHI categories (Address, Contact, Date, Person) using six LLMs, and fine-tuned two PLMs for PHI de-identification on both the synthetic corpus and two real-world datasets. The models were subsequently evaluated on real-world benchmark datasets using a harmonized label set to determine which lexical and semantic characteristics of the training and evaluation corpora correlate with performance degradation in out-of-domain and out-of-distribution (OOD) settings, particularly when models trained exclusively on synthetic notes are applied to real clinical text.

In this paper, we first review related work, then describe the procedure for generating the synthetic corpus. Next, we detail the lexical and semantic similarity measures used to analyze the relationship between corpus properties and model performance. We subsequently present the results, including an ablation study, discuss their implications for OOD de-identification, and summarize the key findings.

## Related work

### PHI de-identification

In the field of NLP, PHI detection is commonly framed as a sequence-labeling task [15–18]. Because manual de-identification is labor-intensive and clinical notes cannot easily be shared, publicly available resources cover only a narrow range of note types and languages [16, 19, 20]. Privacy constraints, heterogeneous documentation styles, and institutional specific label sets make it harder to create training data that are versatile [21–23]. Advanced de-identification approaches consider re-identification risk through measures based on span probabilities or neural classifiers, using optimization to minimize semantic loss while maintaining privacy [24].

### Synthetic clinical text

To mitigate data scarcity, early studies used rule-based substitution, template filling, or data augmentation, however these can lead to limited linguistic variety and weak context specificity [21, 22, 25]. More recent work instead leverages LLMs to generate task-aware, multilingual clinical data, after which the synthetic data can be used to adapt smaller, on-premise models [23, 25]. Nevertheless, LLM-generated data may show fidelity and boundary errors, or hallucinations. Therefore, human-annotated data often remains stronger on real clinical text [25]. Privacy-by-design frameworks such as RecordTwin and relexicalization approaches therefore combine k-anonymisation with controlled context generation to lower leakage risks while preserving realistic entities [1, 26, 27]. However, such privacy-by-design pipelines further remove institution-specific writing styles and PHI patterns, thereby increasing drift from real notes.

### Distributional drift between synthetic and real data

Even with improved generators, models trained on synthetic or out-of-institution corpora often perform worse on real notes because the target distribution differs in entity priors, note structure, language, or annotation policies. Work on distribution shift in NLP, finds shifts within semantics, surface/structural features, or concepts [28–30]. Drift has been measured with statistical equivalence tests, chi-squared tests over discrete features, and representation-based distances to estimate transfer performance without target labels [28, 31, 32]. Additional approaches use calibration- or perplexity-based indicators, and context-aware drift detection with task-specific tolerance regions [33, 34]. Our study follows this line but focuses on comparing lexical and semantic similarity measures between LLM-generated corpora and real clinical datasets to identify those signals that best predict PHI de-identification performance.

## Datasets

### Generated datasets

We constructed the synthetic corpora with a multi-round, LLM generator ensemble and a single LLM-as-judge [35] to balance diversity and quality. The LLM is instructed to generate realistic medical text fragments annotating the target entity type in line with Murugadoss et al. [36]. Other PHI entity types may naturally co-occur but are left unannotated, consistent with how the downstream models are trained and evaluated one entity type at a time [36, 37]. Five generators were used: Meditron3 Qwen2.5 7B [38], Qwen3 8B [39], Llama 3.1 8B Instruct [40], Ministral 8B Instruct [41], and MedGemma-4B Instruct [42]; a slightly larger judge, Qwen3 14B [39], providing each round a quality score (1–5) for each generated sample. An sanity-check for each generation ensured at minimal the structural correctness of the synthetic annotation.

Generation proceeded for 30 rounds with 250 samples per round across all generators. Round 0 was strictly zero-shot; after each subsequent round, we randomly drew $k{=}10$ highly judged samples from the growing pool to serve as few-shot examples in the context for the next round. The judge threshold was set to 3.0 (aggregation: average), and a cap of 10 occurrences per entity string. A negative-sample ratio of 0.7 controlled class balance via two different prompts. The process was executed separately for each entity type, yielding independent pools per class. This iterative multi-round generation procedure follows the self-improvement loop introduced by Wang et al. [43], with the key difference being that our pipeline requires no manually written seed examples as Round 0 is strictly zero-shot. Furthermore, we use high-scoring samples which are selected using an LLM-as-judge. This serves as the quality filter, whose outputs enter the few-shot pool for subsequent rounds, instead of a string similarity metric.

We produced synthetic corpora (syn) in English for four categories: Address, Contact, Date, and Person. Each synthetic dataset (syn_*ADDRESS*_, syn_*CONTACT*_, syn_*DATE*_, syn_*PERSON*_) contains notes of 2–3 sentence ($\approx$ 57–61 tokens/document) with entity counts from 41 K for Person up to 110 K for Address. All syn are provided in three training sizes (1 K, 10 K, 20 K docs) with a fixed 2 K-document test split.

### Existing datasets

To evaluate our models, we use two real and one synthetic corpora. I2B2 [16] represents long-form clinical notes ($\approx$ 60 sentences, 859 tokens/document) which serves the key evaluation benchmark. MultiLeg [44], in contrast, has very short documents ($\approx$ 53 tokens/document) and it is from the legal domain, which we use for both training and evaluation. Ai4Privacy [45] offers the largest dataset with the biggest vocab size (> 55 K), multiple sublabels for all four PHI categories, and is likewise used for training and evaluation. However, the dataset is synthetic, and the methods used to construct it is not released. Table 1 shows the characteristics of all datasets used.

**Table 1:** Statistics of datasets: document length (in tokens), entity counts per type (Address (A), Contact (C), Date (D), Person (P)), train/test splits, vocab size, and total tokens

Dataset	Doc length	P	D	A	C	# Train docs	# Test docs	Vocab size	Total tokens
I2B2	858.85	5451	19670	1247	661	–	514	21249	441450
MultiLeg	53.42	1729	3319	1357	78	1 K/2841	315	7059	168594
Ai4Privacy	35.45	11948	23996	18005	20460	1 K/10 K/20 K	4358	55108	863546
$\textrm{syn}_{\textrm{PERSON}}$	60.29	41476	–	–	–	1 K/10 K/20 K	2000	19167	1326330
$\textrm{syn}_{\textrm{DATE}}$	56.95	–	87492	–	–	1 K/10 K/20 K	2000	16860	1252937
$\textrm{syn}_{\textrm{ADDRESS}}$	61.17	–	–	109931	–	1 K/10 K/20 K	2000	21785	1345782
$\textrm{syn}_{\textrm{CONTACT}}$	61.19	–	–	–	45845	1 K/10K/20 K	2000	18447	1346101

## Methods

In this section, we first describe the training and evaluation of models on the de-identification task. Then we detail the drift metrics (lexical, structural, and semantic) computed for each train-eval corpus pair.

### PHI model training and evaluation

We fine-tuned two English PLMs for token-classification, BioClinicalBERT [46], and DeBerta v3 base [47], on BIO-formatted PHI spans for the four categories. For every training–evaluation configuration, we trained both models with two random seeds (42, 43). The models were fine-tuned for 5 epochs with batch size 64, learning rate $2\times 10^{-5}$, AdamW, and max sequence length 512. Evaluation applied strict entity-level matching to compute precision, recall, and F1. The F1 score was used as the utility signal for drift correlation. Training one model per entity type, rather than a single multi-class tagger, is an established practice in PHI de-identification [36, 37, 48], and is consistent with the sentence-level fine-tuning paradigm on i2b2 by Murugados et al. [36]. Co-occurring PHI spans of other types are treated as non-entity tokens during training. The generalization of our models fine-tuned on the different datasets is assessed empirically in this study through evaluation on held-out data.

### Drift metrics

We assess distributional drift between real and synthetic corpora across three metric families: surface statistics, structural/layout indicators, and semantic similarity in an embedding space. This prevents apparent gains on one view from masking degradation on another and preserves interpretability for downstream use.

#### Surface-level metrics

Lexical shift is measured with Jensen–Shannon divergence (JSD) [49] on unigram and bigram counts; type-level coverage is captured with vocabulary Jaccard similarity [50, 51]. Style is summarized via sentence-length mean/standard deviation and via absolute differences in token-level categories (punctuation, digits). Lexical diversity is profiled through vocabulary entropy, MTLD [52], and Yule’s K [53], complemented by Distinct-1/2 [54] and an explicit 4-gram self-repetition [55, 56] rate to detect template overuse in synthetic text. Readability is compared via Flesch–Kincaid grade (FK) [57–59] differences, and fluency via GPT-2 [60] perplexity [61].

#### Structural metrics

We track line-break density per 100 tokens, the proportion of key–value-style sentences, the fraction of bullet or enumerated openings, the share of non-single-letter ALL-CAPS tokens, and the fraction of tokens with special characters; together these summarize domain-specific formatting conventions.

#### Semantic (embedding-space) metrics

Sentence embeddings from a compact transformer encoder support distributional comparison. We report maximum mean discrepancy [62, 63] (RBF kernel, median bandwidth) and Fréchet distance between Gaussian summaries [64, 65]; both decline as the corpora align. To test semantic coverage, we fit *k*-means on real embeddings, assign synthetic instances to the centroids, and compute JSD [66, 67] over the cluster histogram; the Euclidean distance between embedding means serves as an interpretable effect-size proxy. Detailed definitions are given in the Appendix ‘Drift metrics details’.

## Results

### Models performance

In the following, we describe the performance of the PLMs trained on the real and synthetic datasets for the de-identification task.

#### Cross-dataset performance

To assess how training source and size affect transfer, we report F1 for every train–test pair (Table 2). As expected, each dataset performs best on its own distribution, and scaling Ai4Privacy from 1 K to 20 K training samples improves both its in-distribution scores and its OOD scores on I2B2. Even so, Ai4Privacy models transfer only moderately to MultiLeg for Address/Contact, indicating a domain/style gap. The syn corpora behave as designed, near-perfect on synthetic tests and competitive on I2B2 for Date and Person, but remain weak on clinical-style Address, which seems harder to generalize when generated in isolation. Overall, Table 2 reveals two constraints that will reappear in the OOD analysis: domain similarity matters, and entity types differ in how generalizable they are.

**Table 2:** Cross-dataset perfomance: average F1 scores (across downstream task models and seeds) for models trained on one corpus and evaluated on four target datasets across ADDRESS, CONTACT, DATE, and PERSON

Train dataset	ADDRESS				CONTACT				DATE				PERSON			
	Ai4Pr	I2B2	Multi	Syn	Ai4Pr	I2B2	Multi	Syn	Ai4Pr	I2B2	Multi	Syn	Ai4Pr	I2B2	Multi	Syn
Ai4Pr 1K	0.406	0.236	0.024	0.098	0.514	0.041	0.000	0.179	0.658	0.148	0.137	0.162	0.717	0.128	0.061	0.084
Ai4Pr 10K	0.966	0.368	0.218	0.119	0.995	0.083	0.000	0.441	0.984	0.335	0.403	0.109	0.976	0.601	0.238	0.500
Ai4Pr 20K	0.986	0.361	0.240	0.119	0.999	0.104	0.000	0.442	0.990	0.285	0.323	0.094	0.989	0.644	0.228	0.482
Multi 1K	0.011	0.003	0.065	0.000	0.000	0.050	0.000	0.000	0.094	0.050	0.657	0.007	0.086	0.118	0.416	0.159
Multi 2.8K	0.190	0.225	0.722	0.001	0.000	0.000	0.000	0.000	0.212	0.120	0.960	0.103	0.310	0.241	0.875	0.291
Syn 1K	0.046	0.011	0.020	0.880	0.031	0.019	0.000	0.629	0.127	0.309	0.097	0.879	0.096	0.189	0.270	0.504
Syn 10K	0.090	0.028	0.020	0.989	0.159	0.061	0.000	0.994	0.199	0.413	0.232	0.939	0.305	0.309	0.410	0.835
Syn 20K	0.094	0.027	0.016	0.997	0.201	0.103	0.000	0.998	0.192	0.430	0.232	0.945	0.313	0.304	0.401	0.875

#### OOD performance

**Fig. 2:**
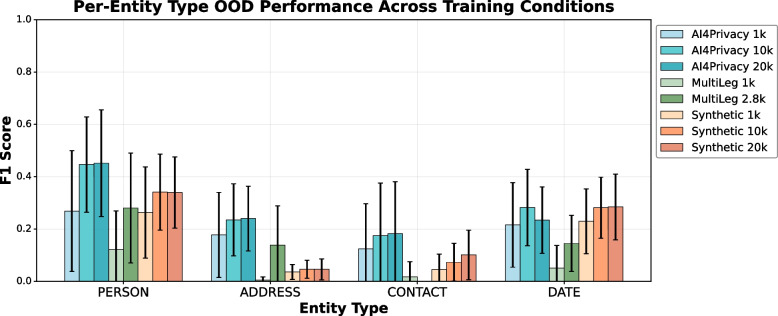
Per-entity OOD F1 scores for models trained on real and synthetic datasets; bars show mean performance and error bars show variability across target datasets

In the OOD setup (Fig. 2), every model is evaluated only on datasets it was not trained on, so the scores reflect genuine cross-corpus generalization. Here, Ai4Privacy is the strongest training set. With 20 K samples, it performs the best for Person, Contact, and Address. The main exception is Date, where our 10K–20 K syn matches Ai4Privacy 10 K–20 K, this could be due to dates being regular enough for focused synthetic generation. MultiLeg, small and legal-domain, stays the weakest signal except for Address, where the syn sets perform worst—consistent with the cross-dataset results. Taken together, the OOD findings reinforce the cross-dataset view: synthetic data performs best for regular entity types (Date, Person), while showing difficulties in heterogeneous entity types, format- and domain-sensitive PHI.

### Drift metrics

To assess the relationship between data shifts and the observed decrease in performance, we computed 24 drift metrics for each entity-specific train-test pair and ranked them by their absolute Pearson correlation (*r*) with $\Delta$F1, defined as the performance difference between best real-trained F1 and mean synthetic-trained F1, such that higher values indicate greater degradation. Values around 0.5 indicate a moderate positive association between higher drift and greater performance degradation. The heatmap in Fig. 4 shows a clear pattern: for most metrics, there is a sharp separation between in-distribution (ID) evaluations (upper part, mostly blue) and OOD evaluations (lower part, mostly red). The strongest predictors are the distributional ones. **JSD Unigram** and **JSD Bigram** (both *r* = 0.54) are almost uniformly red in OOD rows, showing that shifts in token or short-context frequencies are closely tied to F1 drops. The next group, **MTLD Diff** (*r *= 0.53), **Perplexity Diff** (*r *= 0.52), **Flesch–Kincaid Diff** (*r* = 0.51), still marks many OOD cases but with more variation, meaning that diversity and complexity differences do not affect all OOD transfers but are predictive when they occur. Representation-level metrics, **Cluster Drift**, **Embedding Mean Distance**, **MMD** ($\approx$ 0.49–0.50) again label most OOD rows as high drift, confirming that the shift is visible at the embedding level as well. Unlike the above-mentioned metric, **Vocab Overlap** (*r *= – 0.47) appears inverted because higher overlap means lower drift, but it follows the same ID/OOD split. However, there are no clear distinguishing patterns for style-based features (capitalization, punctuation, special characters).

## Ablation study

We conducted three ablation studies to quantify which components of the synthetic-data pipeline affect downstream PHI tagging under OOD evaluation. First, we trained on subsets with LLM-as-judge quality scores $\ge$ 3.0, $\ge$ 4.0, and $=$ 5.0. Second, we trained models on mixed real (MultiLeg) and synthetic (syn) datasets, with synthetic data proportions ranging from 0% to 100%. Third, we trained on synthetic data from each of our five generator models.

**Fig. 3:**
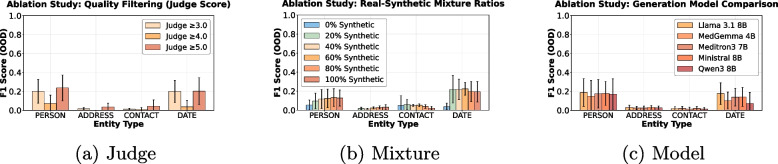
Out-of-distribution performance for all three ablation studies: **a** filtering data by judge score, **b** varying real-synthetic mixing ratios, **c** comparing generation models

**Fig. 4:**
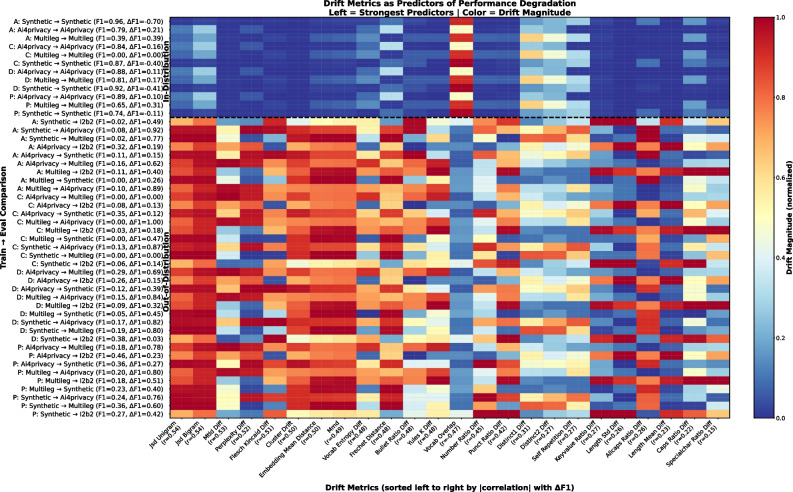
Cross-dataset drift as a predictor of OOD performance. Rows are train$\rightarrow$test configurations for 4 PHI types, Address (A), Contact (C), Date (D) and Person (P), with columns showing drift metrics sorted by |correlation| with $\Delta$F1; drift magnitudes are averaged over training sizes (1k, 10k, 20k) for visual clarity, while Spearman r values are computed over all individual training-size configurations. Early distributional/embedding metrics clearly separate in-distribution from out-of-distribution evaluations

**Fig. 5:**
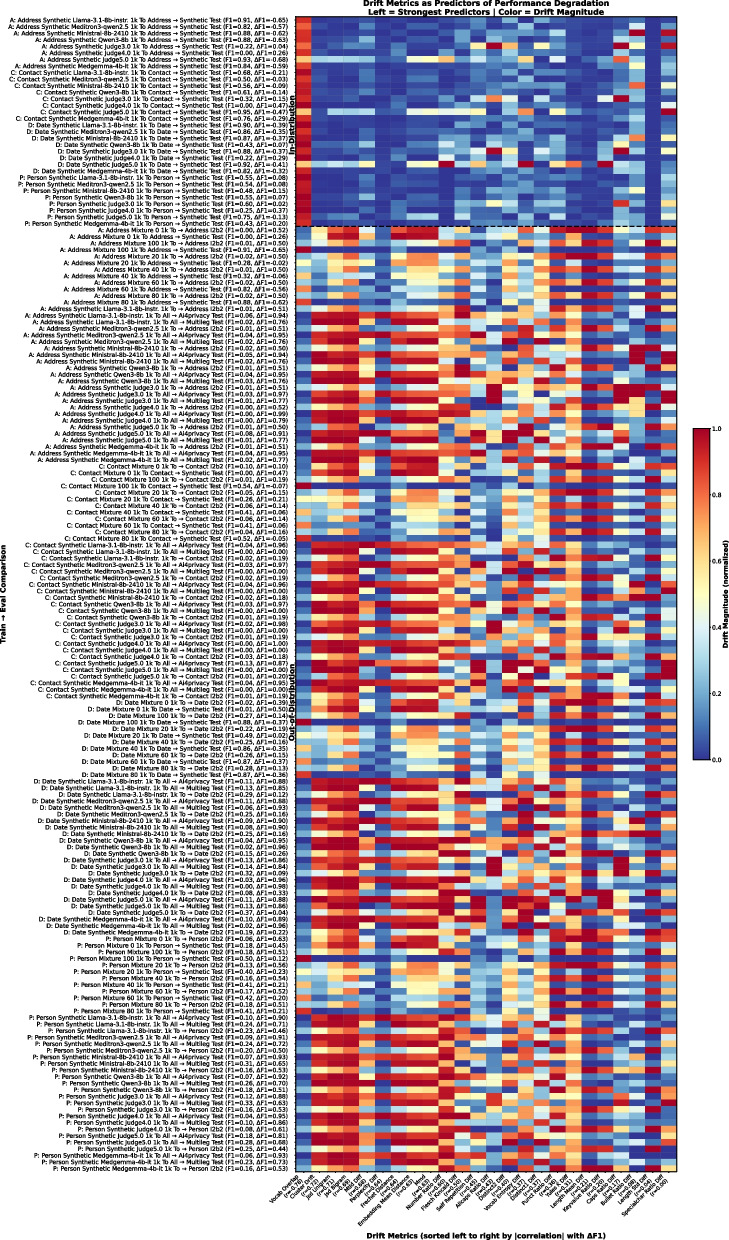
Ablation-level Cross-dataset drift as a predictor of OOD degradation. Rows are train$\rightarrow$test configurations for 4 PHI types Address (A), Contact (C), Date (D) and Person (P); columns are drift metrics sorted by |correlation| with $\Delta$F1. Early distributional/embedding metrics clearly separate in-distribution (blue) from out-of-distribution (red) evaluations.

Across all three ablation experiments, scores are reported as OOD F1, averaged over the target datasets and per entity type. In the quality-filtering setting (Fig. 3a), Person and Date benefit the most from high-scoring synthetic instances ($\ge$ 5.0), whereas Address and Contact show minimal changes. In the mixtures (Fig. 3b), models trained with some mixture of real and synthetic data (20–60%) obtain higher OOD F1 than those trained only on real data for all four entity types, with the largest relative gains for Date; performance declines or stagnates when moving to > 80% synthetic data. In the generator comparison (Fig. 3c), OOD F1 values are largely similar across the five LLMs; small differences are visible for Person and Date. In the ablation drift heatmap Fig. 5 in Appendix 3, the most predictive measures, in order, are Vocab Overlap (*r* = − 0.78), Cluster Drift (*r* = 0.72), JSD Unigram (*r* = 0.71), and JSD Bigram (*r* = 0.69), showing that representation-level and n-gram distribution shifts remain the strongest signals, but that vocab overlap becomes more informative than in the main analysis. Metrics that capture lexical diversity or readability show weaker correlations. Unlike the main analysis, where correlations are moderate due to large cross-domain gaps, the ablation setting yields stronger correlations (Fig. 4).

## Discussion

### Drift metrics as OOD performance proxies

The results demonstrate that corpus-level distributional and embedding-space metrics can serve as indicators of OOD performance degradation in PHI de-identification without requiring access to target labels. Building on prior work characterizing distribution shift in the NER task [28], we show that such metrics are moderately correlated with downstream task performance in the clinical synthetic-to-real setting. Notably, surface-stylistic features, despite capturing perceptible differences between synthetic and real text, show little consistent predictive value, suggesting that transfer difficulty is governed by token-distributional and semantic-coverage gaps.

### Entity-invariant predictive capacity of drift metrics

Despite variation in OOD transfer performance across PHI categories, with Address exhibiting the most performance degradation and Date the most generalisability, the discriminative capacity of drift metrics remains relatively consistent across all four entity types. This consistency across entity types suggests entity-invariant predictive capacity of the identified drift metrics.

### Implications for synthetic data pipelines

These findings suggest that drift-based screening can complement quality filtering as a means of estimating transfer risk prior to model training, without requiring access to annotated target data. The stronger predictive capacity observed in the ablation setting suggests that lexical coverage metrics are particularly discriminative when distributional differences are constrained to synthetic pipeline variations rather than cross-domain shifts.

## Conclusion

This exploratory study set out to understand how measurable drift between clinical synthetic and real clinical text relates to downstream performance, using PHI de-identification as a use case. We found a small set of distributional and representation-based metrics that are indicative of a model’s OOD performance degradation on downstream tasks, such as PHI de-identification. The ablation results further suggested that synthetic data is most useful as an additive signal to real corpora, rather than as a full substitute. However, these observations are specific to our generation pipeline, our datasets, harmonized label space, and English setting, and should be validated on other synthetic data generation approaches, data from additional institutions, languages, and tasks.

## Figures and Tables

**Fig. 6:**
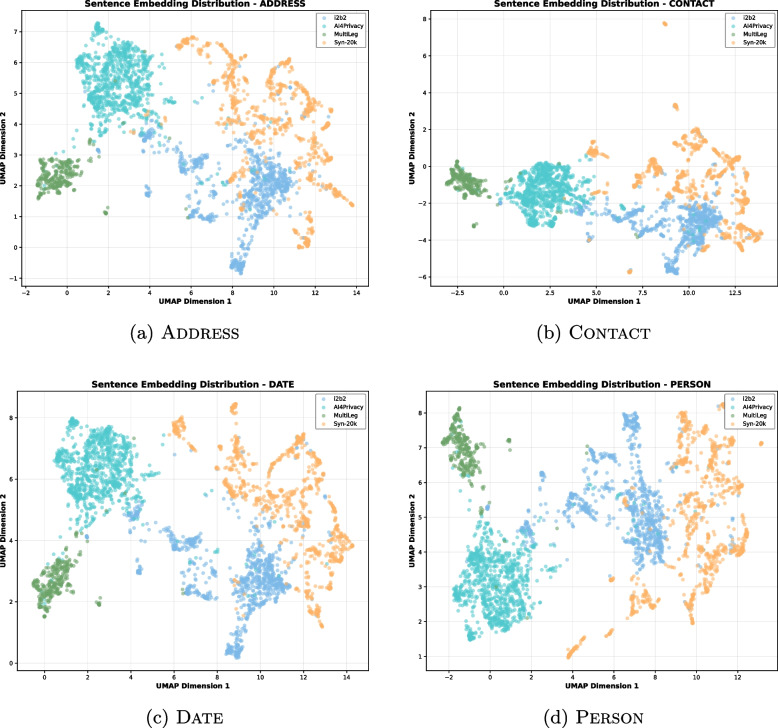
UMAP projection of sentence-level embeddings per PHI category. Each subplot shows the embedding distributions of i2b2 (green), ai4privacy (teal), multileg (blue), and synthetic 20 k (orange) notes for **a** Address, **b** Contact, **c** Date, and **d** Person. Synthetic data is closest to ai4privacy for Date and Person, while Address and Contact exhibit clearer separation between real and synthetic sources

## Data Availability

The data generated for this study is available at: https://github.com/IDSIA-NLP/measuring-the-gap.

## Appendix 1: Harmonization

This appendix section documents how heterogeneous entity labels from the four source datasets were mapped into a unified schema covering Person, Date, Contact, and Address to enable joint modeling and evaluation, as seen in Table 3

**Table 3:** Label harmonization across the four datasets, showing original dataset-specific tags and their mapping to the unified entity set PERSON, DATE, CONTACT, ADDRESS. And the number of appearances of the original tags in the corresponding datasets

Entity	i2b2		MultiLeg		AI4Privacy	
	Original tag	Count	Original tag	Count	Original tag	Count
ADDRESS	CITY	347	LOC	1357	BUILDINGNUM.	1494
	COUNTRY	130	–	–	CITY	2326
	STATE	206	–	–	COUNTY	2472
	STREET	416	–	–	SECONDARYAD.	3139
	ZIP	148	–	–	STATE	2678
	–	–	–	–	STREET	3255
	–	–	–	–	ZIPCODE	2641
CONTACT	EMAIL	5	IDNUM	78	EMAIL	13155
	FAX	12	–	–	PHONENUM.	7305
	PHONE	644	–	–	–	–
PERSON	DOCTOR	3642	PER	1729	FIRSTNAME	5966
	PATIENT	1809	–	–	FULLNAME	3391
	–	–	–	–	LASTNAME	1535
	–	–	–	–	MIDDLENAME	1056
DATE	DATE	19670	DATE	3319	DATE	13005
	–	–	–	–	DOB	6550
	–	–	–	–	TIME	4441

## Appendix 2: Model details

This appendix summarizes the models and training configurations used in our experiments. Table 4 lists the generative pipeline models, distinguishing five generators from a single judge model and indicating the number of rounds and samples used in the data-generation loop. Table 5 reports the supervised fine-tuning setup for the discriminative models (Bio_ClinicalBERT and DeBERTa v3 base), including sequence length, batch size, and optimization hyperparameters.

**Table 4:** Generative pipeline models used for synthetic data creation, showing generator and judge roles, model identifiers, sampling settings, and per-round workload

Role	Model (full ID)	Short name	Size	Max tokens	Temp	Top-p	Top-k	Samples/round	Rounds
Generator	OpenMeditron/Meditron3-Qwen2.5-7B	Meditron3-Qwen2.5	7B	2048	default	default	default	250	30
Generator	Qwen/Qwen3-8B	Qwen3-8B	8B	2048	0.7	0.8	20	250	30
Generator	meta-llama/Llama-3.1-8B-Instruct	Llama-3.1-8B-Instr.	8B	2048	default	default	default	250	30
Generator	Ministral-8B-Instruct-2410	Ministral-8B-2410	8B	2048	default	default	default	250	30
Generator	google/medgemma-4b-it	MedGemma-4B-IT	4B	2048	default	default	default	250	30
Judge	Qwen/Qwen3-14B	Qwen3-14B (judge)	14B	2048	0.7	0.8	20	250	30

**Table 5:** Model configurations for discriminative baselines (Bio_ClinicalBERT and DeBERTa v3 base), including input length, batch size, number of epochs, and optimization parameters

Model name	Model type	Max seq length	Batch size	Epochs	Learning rate	Warmup ratio	Weight decay
emilyalsentzer/Bio_ClinicalBERT	BERT	512	64	5	2.0$\times 10^{-5}$	0.1	0.01
microsoft/deberta-v3-base	DeBERTa	512	64	5	3.0$\times 10^{-5}$	0.1	0.01

### Drift metrics details

This appendix lists the drift metrics we use to compare corpora. Metrics are grouped into (i) surface/lexical, (ii) structural/layout, and (iii) semantic/embedding-space. Unless noted, every metric can be run on any pair of corpora: real vs synthetic, real vs real, or synthetic vs synthetic. For a metric (m) we report its value on corpus A ($m_{\text {A}}$), on corpus B ($m_{\text {B}}$), and the absolute difference $|m_{\text {A}} - m_{\text {B}}|$ so departures are directly auditable. Divergence-style metrics are reported so that lower values mean closer alignment, unless stated otherwise. In the following, we refer to the case of real vs synthetic.

#### Surface-level (lexical) metrics.

**Jensen–Shannon divergence (unigram/bigram).** Let *P* and *Q* be the empirical *n*-gram distributions from the real and synthetic corpora, and $M = \frac{1}{2} (P+Q)$. We compute

$$\begin{aligned} \textrm{JSD}(P,Q) = \frac{1}{2} \textrm{KL}(P \,\Vert \, M) + \frac{1}{2} \textrm{KL}(Q \,\Vert \, M) \end{aligned}$$

with a small $\varepsilon$-smoothing over the union of vocabularies; $n{=}1$ (unigram) captures token choice and $n{=}2$ (bigram) captures local collocations. Lower values mean closer lexical match.

**Vocabulary overlap (Jaccard similarity).** With $V_{\text {real}}$ and $V_{\text {syn}}$ the token sets (case-folded), we report

$$\begin{aligned} J = \frac{|V_{\text {real}} \cap V_{\text {syn}}|}{|V_{\text {real}} \cup V_{\text {syn}}|}, \end{aligned}$$

which highlights type-level gaps independently of frequency.

**Vocabulary entropy.** For each corpus we form the empirical token distribution *p*(*w*) and compute

$$\begin{aligned} H = - \sum \limits _{w} p(w)\log p(w), \end{aligned}$$

then report $|H_{\text {real}} - H_{\text {syn}}|$. Higher entropy indicates broader lexical variety.

**Lexical diversity (MTLD, Yule’s K).** We report MTLD (larger $\Rightarrow$ more diverse, less length-sensitive) together with Yule’s K (smaller $\Rightarrow$ more diverse). Joint inspection distinguishes genuine variety from short-range repetition.

**Distinct-*****n*** **(Distinct-1/2).** For $n \in \{1,2\}$ we compute

$$\begin{aligned} \text {Distinct-}n = \frac{\#\text {unique}\ n\text {-grams}}{\#\text {all}\ n\text {-grams}}, \end{aligned}$$

which summarizes within-corpus repetition; higher values indicate less degeneracy.

**Self-repetition rate (repeated** ***n*****-grams).** Using default $n{=}4$, we measure the proportion of *n*-grams that occur more than once in the same corpus. Elevated values in synthetic data are typical of template reuse or mode collapse.

**Sentence-length statistics.** From sentence token counts we compute mean ($\mu$) and standard deviation ($\sigma$) per corpus and report $|\mu _{\text {real}} - \mu _{\text {syn}}|$ and $|\sigma _{\text {real}} - \sigma _{\text {syn}}|$. This captures verbosity/terseness drift that affects readability and downstream sequence models.

**Character-category ratios.** We separately compare (i) punctuation-bearing tokens, (ii) digit-containing tokens, and (iii) initially capitalized tokens, each as a proportion of all tokens. Absolute differences flag shifts in numeracy, list-like style, or proper-noun density.

**Readability (Flesch–Kincaid).** We compute the Flesch–Kincaid grade level for each corpus and take the absolute difference to quantify stylistic/accessibility drift not captured by *n*-gram counts.

**Language-model perplexity.** Perplexity under a fixed pretrained LM (here GPT-2) is

$$\begin{aligned} \textrm{PPL} = \exp \!\left( -\frac{1}{N} \sum \limits _{i=1}^{N} \log p(w_i)\right) , \end{aligned}$$

where $p(w_i)$ is the model probability on held-out text.

#### Structural (layout/format) metrics

**Line-break density.** We compute the average number of explicit line-break markers per 100 tokens. Divergences point to different paragraphing or wrapping conventions that affect rendering and segmentation.

**Key–value pattern ratio.** We measure the proportion of sentences that match common key–value or delimiter patterns (e.g., Name: John, A=B). Matching this rate is important for logs/forms-like text where downstream extraction expects these cues.

**Bullet/enumeration ratio.** This is the fraction of sentences whose first token is a bullet symbol or an ordinal with a trailing period; it indexes list-oriented style typical of instructions or technical notes. Underrepresentation in synthetic data signals structural undergeneration.

**All-caps token ratio.** We compute the share of multi-letter ALL-CAPS tokens, excluding single-letter tokens. Differences indicate changes in headings, acronym usage, or emphasis conventions.

**Special-character ratio.** The proportion of tokens containing characters such as @#$%â & approximates the incidence of code-like, markup, or templated content. In our setup, this serves as the light-weight substitute for explicit “code-snippet” detection.

#### Semantic (embedding-space) metrics.

**Maximum mean discrepancy (MMD).** Let $\{x_i\}_{i=1}^{n}$ and $\{y_j\}_{j=1}^{m}$ be sentence embeddings for real and synthetic text, and let $k(\cdot ,\cdot )$ be the RBF kernel with bandwidth from the median heuristic. We use the biased estimator

$$\begin{aligned} \textrm{MMD}^2 = \frac{1}{n^2}\sum \limits _{i,i'} k(x_i,x_{i'}) + \frac{1}{m^2}\sum \limits _{j,j'} k(y_j,y_{j'}) - \frac{2}{nm}\sum \limits _{i,j} k(x_i,y_j), \end{aligned}$$

and report $\textrm{MMD} = \sqrt{\textrm{MMD}^2}$. Values close to 0 indicate well-aligned embedding distributions.

**Fréchet distance (Gaussian summaries).** We approximate the two embedding sets by Gaussians $(\mu _r,\Sigma _r)$ and $(\mu _s,\Sigma _s)$ and compute

$$\begin{aligned} d^2 = \Vert \mu _r - \mu _s\Vert _2^2 + \textrm{Tr}\left( \Sigma _r + \Sigma _s - 2(\Sigma _r \Sigma _s)^{1/2}\right) , \end{aligned}$$

which penalizes both mean shifts and covariance mismatches, analogous to FID.

**Embedding-mean distance.** As an interpretable effect-size proxy we report

$$\begin{aligned} \Vert \mu _r - \mu _s\Vert _2, \end{aligned}$$

i.e., the Euclidean distance between the corpus-level mean embeddings. This is easy to monitor alongside MMD/Fréchet.

**Cluster-coverage divergence.** We run *k*-means on real embeddings to obtain cluster proportions *p*(*c*) and assign synthetic embeddings to the same centroids to obtain *q*(*c*). Drift is summarized by $\textrm{JSD}(p,q)$ (as above).

## Appendix 3: Ablation

This appendix subsection provides extended details ablation experiments.

**Table 6:** Detailed ablation results with per-label and overall metrics for fine-grained model settings

Train dataset	ADDRESS				CONTACT				DATE				PERSON			
	Ai4Pr	I2B2	Multil	Syn	Ai4Pr	I2B2	Multil	Syn	Ai4Pr	I2B2	Multil	Syn	Ai4Pr	I2B2	Multil	Syn
Mixture 0 1K	0.000	0.000	0.000	0.000	0.000	0.101	0.000	0.000	0.052	0.025	0.808	0.006	0.043	0.065	0.499	0.175
Mixture 100 1K	0.054	0.011	0.028	0.910	0.034	0.010	0.000	0.543	0.116	0.272	0.072	0.880	0.071	0.185	0.251	0.502
Mixture 20 1K	0.019	0.018	0.012	0.283	0.070	0.048	0.000	0.258	0.220	0.219	0.710	0.490	0.062	0.132	0.508	0.397
Mixture 40 1K	0.014	0.014	0.050	0.322	0.044	0.057	0.000	0.411	0.193	0.246	0.733	0.862	0.077	0.156	0.490	0.410
Mixture 60 1K	0.030	0.018	0.012	0.817	0.044	0.060	0.000	0.412	0.188	0.263	0.702	0.872	0.076	0.172	0.379	0.423
Mixture 80 1K	0.040	0.020	0.025	0.877	0.038	0.041	0.000	0.519	0.113	0.281	0.189	0.872	0.083	0.181	0.284	0.412
Synthetic Judge3.0 1K	0.027	0.008	0.012	0.218	0.023	0.011	0.000	0.319	0.133	0.324	0.145	0.882	0.115	0.161	0.326	0.604
Synthetic Judge4.0 1K	0.000	0.000	0.000	0.000	0.000	0.025	0.000	0.000	0.031	0.082	0.000	0.216	0.042	0.077	0.100	0.253
Synthetic Judge5.0 1K	0.084	0.012	0.014	0.935	0.128	0.006	0.000	0.946	0.114	0.366	0.126	0.917	0.181	0.249	0.281	0.755
Synthetic Llama-3.1-8B-Instr. 1K	0.057	0.008	0.024	0.912	0.041	0.015	0.000	0.679	0.112	0.289	0.129	0.900	0.095	0.227	0.244	0.546
Synthetic MedGemma-4B-IT 1K	0.043	0.008	0.020	0.844	0.045	0.013	0.000	0.758	0.102	0.188	0.019	0.823	0.060	0.158	0.231	0.429
Synthetic Meditron3-Qwen2.5 1K	0.042	0.009	0.021	0.825	0.030	0.015	0.000	0.504	0.110	0.251	0.056	0.858	0.085	0.195	0.240	0.545
Synthetic Ministral-8B-2410 1K	0.054	0.019	0.022	0.878	0.038	0.024	0.000	0.561	0.092	0.250	0.082	0.874	0.069	0.160	0.307	0.476
Synthetic Qwen3-8B 1K	0.044	0.008	0.029	0.884	0.032	0.013	0.000	0.613	0.044	0.151	0.020	0.434	0.074	0.182	0.257	0.554

### UMAP visualization

This appendix subsection visualizes the four datasets (two real, one synthetic, one synthetic) per label using UMAP to assess domain similarity and to verify that synthetic/real data occupy a comparable feature space to real data, provided in Fig. 6.

## Materials availability

All materials generated during this study will be made available upon request.

## Code availability

All code used for the analyses is available at: https://github.com/IDSIA-NLP/measuring-the-gap.
